# Maximal Exercise Testing Using the Incremental Shuttle Walking Test Can Be Used to Risk-Stratify Patients with Pulmonary Arterial Hypertension

**DOI:** 10.1513/AnnalsATS.202005-423OC

**Published:** 2021-01

**Authors:** Robert A. Lewis, Catherine G. Billings, Judith A. Hurdman, Ian A. Smith, Matthew Austin, Iain J. Armstrong, Jennifer Middleton, Alexander M. K. Rothman, John Harrington, Neil Hamilton, Abdul G. Hameed, A. A. Roger Thompson, Athanasios Charalampopoulos, Charlie A. Elliot, Allan Lawrie, Ian Sabroe, Jim M. Wild, Andrew J. Swift, Robin Condliffe, David G. Kiely

**Affiliations:** ^1^ Sheffield Pulmonary Vascular Disease Unit, Sheffield Teaching Hospitals National Health Service Foundation Trust, Royal Hallamshire Hospital, Sheffield, United Kingdom; ^2^ Department of Infection, Immunity and Cardiovascular Disease, University of Sheffield, Medical School, Sheffield, United Kingdom; and; ^3^ Insigneo Institute for *in silico* Medicine, University of Sheffield, Sheffield, United Kingdom

**Keywords:** exercise testing, risk stratification, outcome

## Abstract

**Rationale:** Exercise capacity predicts mortality in pulmonary arterial hypertension (PAH), but limited data exist on the routine use of maximal exercise testing.

**Objectives:** This study evaluates a simple-to-perform maximal test (the incremental shuttle walking test) and its use in risk stratification in PAH.

**Methods:** Consecutive patients with pulmonary hypertension were identified from the ASPIRE (Assessing the Spectrum of Pulmonary hypertension Identified at a REferral centre) registry (2001–2018). Thresholds for levels of risk were identified at baseline and tested at follow-up, and their incorporation into current risk stratification approaches was assessed.

**Results:** Of 4,524 treatment-naive patients with pulmonary hypertension who underwent maximal exercise testing, 1,847 patients had PAH. A stepwise reduction in 1-year mortality was seen between levels 1 (≤30 m; 32% mortality) and 7 (340–420 m; 1% mortality) with no mortality for levels 8–12 (≥430 m) in idiopathic and connective tissue disease–related PAH. Thresholds derived at baseline of ≤180 m (>10%; high risk), 190–330 m (5–10%; intermediate risk), and ≥340 m (<5%; low risk of 1-yr mortality) were applied at follow-up and also accurately identified levels of risk. Thresholds were incorporated into the REVEAL (Registry to Evaluate Early and Long-Term Pulmonary Arterial Hypertension Disease Management) 2.0 risk score calculator and French low-risk approach to risk stratification, and distinct categories of risk remained.

**Conclusions**: We have demonstrated that maximal exercise testing in PAH stratifies mortality risk at baseline and follow-up. This study highlights the potential value of the incremental shuttle walking test as an alternative to the 6-minute walking test, combining some of the advantages of maximal exercise testing and maintaining the simplicity of a simple-to-perform field test.

Pulmonary arterial hypertension (PAH) is a life-shortening condition, and risk stratification is recommended to guide treatment decisions. Exercise limitation is an early presenting symptom in PAH, and measures of exercise capacity are typically severely reduced (1, 2). Exercise testing is recommended as part of a multiparameter assessment in the European Society of Cardiology (ESC)/European Respiratory Society (ERS) and REVEAL 2.0 risk scores and has been frequently used as an endpoint in clinical trials (3, 4).

The 6-minute-walk test (6MWT) is the most widely used exercise test in pulmonary hypertension and is inexpensive and simple to perform (5, 6). Absolute 6MWT distance (6MWD) correlates with hemodynamic parameters in idiopathic PAH (IPAH) and predicts survival at baseline and follow-up (7–10). Nonetheless, there are concerns about a ceiling effect above a distance of 450 m, and younger patients with severe disease may walk beyond 500 m (11–14). In addition, the improvement of 6MWD in response to treatment has not been found to be independently prognostic in PAH (13, 15). Cardiopulmonary exercise testing (CPET) is a maximal test and provides comprehensive evaluation of multiorgan response to physical effort. Parameters from CPET are associated with prognosis in PAH, but its use in routine clinical practice may be limited by the cost, complexity, and duration of procedure (16).

The incremental shuttle walking test (ISWT) is an alternative maximal test for assessing patients with PAH and is used in other forms of cardiac and respiratory disease (17–19). Previous studies have demonstrated correlation between ISWT distance (ISWD) and hemodynamic parameters at right heart catheterization (RHC), and have confirmed that baseline and follow-up distances predict survival in PAH (20). The ISWT has a potential advantage over the 6MWT in that it does not suffer from a ceiling effect, potentially allowing better assessment in patients who are younger or have less severe disease (20, 21). Given the recognized limitations of the 6MWT, we sought to evaluate whether the ISWT could be used to risk-stratify patients with PAH. The aim of this study was to assess whether thresholds could be identified for the ISWT and implemented into widely used risk stratification scores.

## Methods

Patients were identified from the ASPIRE (Assessing the Spectrum of Pulmonary hypertension Identified at a REferral centre) registry and had pulmonary hypertension diagnosed between first February 2001 and 31st May 2018. Patients underwent multimodality assessment as previously described (22). Data were collected prospectively, and patients were required to have an ISWT performed at time of pulmonary hypertension diagnosis before the commencement of PAH therapy. Patients with idiopathic, drug, and heritable PAH were grouped and are referred to hereafter as IPAH. Patients with IPAH and PAH related to connective tissue disease (PAH-CTD) typically represent the majority of patients with PAH in registry studies, and patients in these groups were therefore used to establish and test thresholds (23–25). Thresholds for low, intermediate. and high risk of 1-year mortality were defined as <5%, 5–10%, and >10%, respectively, and were identified in incident, treatment-naive patients based on 1-year mortality (or need for lung transplantation) for each level. Thresholds were evaluated at follow-up, which was defined as the first reassessment beyond 90 days after commencing treatment. For the 6MWT, it is recognized that younger patients with severe disease may walk low-risk distances of >500 m; therefore, in the present study, a sensitivity analysis was performed on patients aged <50 years to assess whether the thresholds remained valid in stratifying risk in younger patients (12, 26).

### The ISWT

The ISWT was undertaken as described by Singh and colleagues (27) and as part of the standard patient evaluation. Patients complete a 10-m length keeping in time to an external audible signal. Level one consists of three lengths (30 m), and each subsequent level adds one extra length to the preceding level. The initial speed is a slow walk (0.50 m/s), increasing incrementally every level to a maximum speed of 2.37 m/s at level 12. Each level takes 1 minute to complete, and the test finishes at the end of level 12, a distance of 1,020 m. The patient continues until they are too breathless or unable to keep up with the required pace (*see* Table 1 for details of walking speeds). Patients who were unable to perform an ISWT because of breathlessness were ascribed an ISWD of 0 m.

**Table 1.** Levels of the incremental shuttle walking test and associated mortality

			*N* (*%*)	1-Yr Mortality (*%*)		
ISWT Level*	Distance (*m*)	Speed (*m/s*)	IPAH and PAH-CTD	IPAH and PAH-CTD	IPAH	PAH-CTD
Level 1	0–30	0.50	267 (22)	31.8	23.4	40.8
Level 2	40–70	0.67	206 (17)	18.5	11.0	24.3
Level 3	80–120	0.84	196 (16)	15.3	12.4	17.8
Level 4	130–180	1.01	172 (14)	14.5	15.2	14.2
Level 5	190–250	1.18	137 (11)	9.5	4.5	14.1
Level 6	260–330	1.35	110 (9)	4.5	5.5	3.6
Level 7	340–420	1.52	69 (6)	1.4	2.3	0
Level 8	430–520	1.69	52 (4)	0	0	0
Level 9	530–630	1.86	18 (2)	0	0	0
Level 10	640–750	2.03	9 (1)	0	0	0
Level 11	760–880	2.20	2 (0)	0	0	—
Level 12	890–1,020	2.37	2 (0)	0	0	—

*Definition of abbreviations*: IPAH = idiopathic pulmonary arterial hypertension; ISWT = incremental shuttle walking test; PAH-CTD = pulmonary arterial hypertension related to connective tissue disease.

* Each level has a duration of 1 minute.

### Mortality Data

Mortality data were obtained from the nationally reported National Health Service Personal Demographics Service, which is updated when a death is registered in the United Kingdom, and transplant data were obtained from local databases. Patients who emigrated (*n* = 3) were excluded from the study as were patients not linked to a record on the Personal Demographics Service (*n* = 2). The outcome assessed was transplant-free survival, and the census date was May 31, 2019, providing at least 1 year of follow-up for all patients.

### Statistical Analysis

Statistical analysis was performed using SPSS version 25 (IBM) and GraphPad Prism version 8. Continuous data were displayed as either mean ± standard deviation or median (first quartile, third quartile) for nonparametric data. Demographics were compared using paired and unpaired *t* tests for parametric data, and Wilcoxon signed-rank and Mann-Whitney *U* tests were used for nonparametric data. Frequencies were compared using χ^2^. A *P* value of less than 0.05 was considered significant. Kaplan-Meier survival curves were compared using log-rank χ^2^. From receiver operating characteristic analysis, a *C*-statistic was produced to compare variations on risk scores. When ISWT levels demonstrated 1-year mortality of 0%, these levels were combined for Kaplan-Meier analysis and correlation with hemodynamics.

### Ethics

Approval by the relevant ethics committee was sought and gained (STH14169, National Health Service Research Ethics Committee 16/YH/0352), and written consent was waived.

## Results

**Figure 1.**
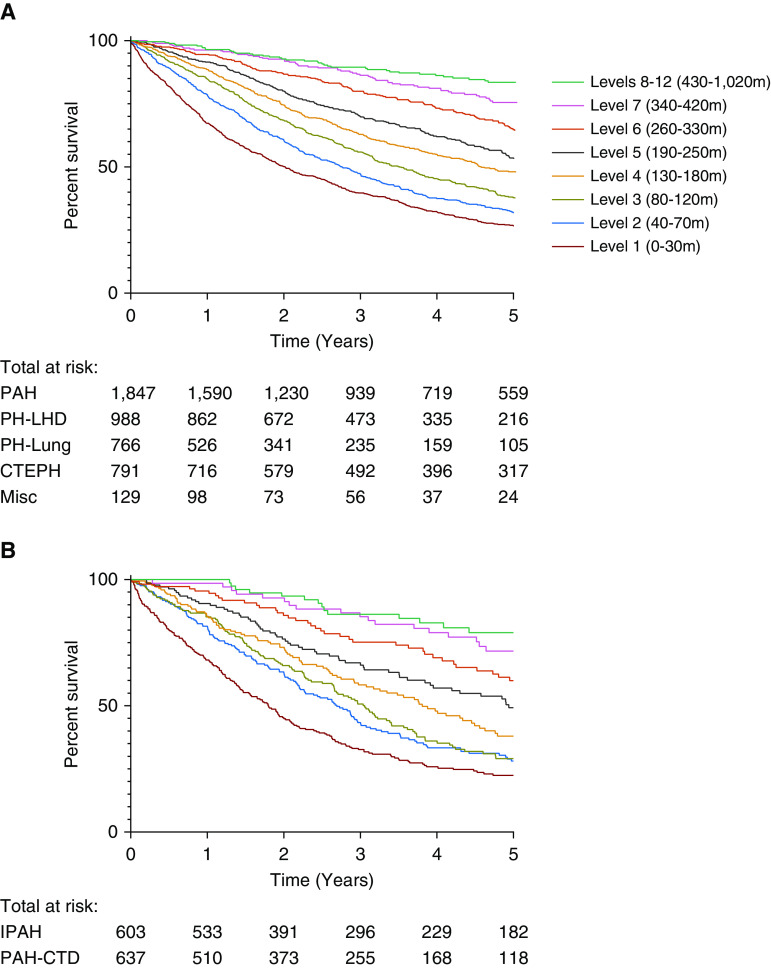
Kaplan-Meier survival curves for (*A*) incremental shuttle walking test distance in all pulmonary hypertension and (*B*) incremental shuttle walking test distance in idiopathic pulmonary arterial hypertension and pulmonary arterial hypertension due to connective tissue disease. CTEPH = chronic thromboembolic pulmonary hypertension; IPAH = idiopathic pulmonary arterial hypertension; LHD = left heart disease; PAH = pulmonary arterial hypertension; PAH-CTD = pulmonary arterial hypertension due to connective tissue disease; PH = pulmonary hypertension.

A total of 4,524 treatment-naive patients with pulmonary hypertension who had undergone ISWT at the time of diagnosis were identified from the ASPIRE registry. Baseline characteristics for different forms of pulmonary hypertension are displayed in Table E1 in the online supplement. Of these, 1,240 had either IPAH or PAH-CTD (Table 2). Kaplan-Meier analysis for ISWD in all forms of pulmonary hypertension and for IPAH/PAH-CTD at baseline are displayed in Figure 1.

**Table 2.** Baseline demographics in patients with IPAH and PAH-CTD

	IPAH & PAH-CTD	IPAH	PAH-CTD
*n*	1,240	603	637
Sex, F, %	71	61	80
Age, yr	64 (53, 72)	62 (47, 72)	66 (57, 73)
WHO FC I, %	0	0	0
WHO FC II, %	13	13	13
WHO FC III, %	63	59	67
WHO FC IV, %	23	27	19
BMI, kg/m^2^	27 (23, 31)	28 (24, 33)	26 (22, 30)
mRAP, mm Hg	9 (6, 14)	11 (7, 15)	8 (5, 12)
mPAP, mm Hg	48 (40, 56)	52 (46, 60)	43 (34, 51)
PAWP, mm Hg	10 (8, 13)	11 (8, 13)	10 (7, 12)
PVR, WU	9.1 (5.7, 13.2)	10.5 (7.8, 14.5)	7.3 (4.7, 11.7)
SvO_2_, %	63 (56, 69)	61 (55, 67)	65 (58, 71)
Cardiac output, L/min	4.3 (3.2, 5.1)	4.0 (3.2, 5.0)	4.4 (3.4, 5.3)
Cardiac index, L/min/m^2^	2.4 (1.9, 2.9)	2.2 (1.8, 2.7)	2.6 (2.0, 3.1)
ISWD, m	110 (40–220)	120 (40–260)	100 (40–195)
			
Treatment, %			
None or calcium channel blocker	2	3	1
Oral mono	33	27	38
Combo oral	42	43	41
Prostanoid ± oral	23	27	20

*Definition of abbreviations*: BMI = body mass index; IPAH = idiopathic pulmonary arterial hypertension; ISWD = incremental shuttle walking test distance; mPAP = mean pulmonary arterial pressure; mRAP = mean right atrial pressure; PAH-CTD = pulmonary arterial hypertension related to connective tissue disease; PAWP = pulmonary arterial wedge pressure; PVR = pulmonary vascular resistance; SvO_2_ = mixed venous oxygen saturations; WHO FC = World Health Organization functional class.

Continuous data were nonparametric and are presented as median (first quartile, third quartile).

### IPAH and PAH-CTD

Incident, treatment-naive patients with IPAH (*n* = 603) had significant disease at RHC, with a median mean pulmonary arterial pressure of 52 mm Hg, pulmonary vascular resistance (PVR) of 10.5 WU, and cardiac index of 2.2 L/min/m^2^. Patients with PAH-CTD (*n* = 637) had a median mean pulmonary arterial pressure (mPAP) of 43 mm Hg, PVR of 7.3 WU, and cardiac index of 2.6 L/min/m^2^. The majority of patients received either combination oral treatment or treatment including a prostanoid.

Within 1 year of diagnosis, 197 patients (15.4%) with IPAH and PAH-CTD had died or undergone transplantation. Levels of the ISWT demonstrated an inverse relationship with risk of 1-year mortality (Table 1). Patients who walked 0–30 m had a 1-year mortality of 32%. A stepwise reduction in mortality percentage was seen at each level until a distance of ≥430 m, for which there was a 0% mortality. Assignment of risk categories required concordance for both IPAH and PAH-CTD (Table 1). A high risk of 1-year mortality (>10%) was therefore defined as a distance of ≤180 m, low risk (<5%) was defined as an ISWD ≥340 m, and intermediate risk was defined as an ISWD of 190–330 m. Corresponding values for cardiac magnetic resonance imaging and pulmonary hemodynamic parameters are displayed in Table 3. A stepwise reduction in right ventricular end-systolic volume index (percentage predicted) was seen with each level of the ISWT. When comparing baseline hemodynamic parameters between patients completing level 1 (≤30 m) and level 2 (40–70 m) of the ISWT, there were significant differences in mean right atrial pressure, cardiac index, and mixed venous oxygen saturation (*P* < 0.05 for all).

**Table 3.** Association between ISWT level and hemodynamic and cardiac MRI parameters

ISWT Level	*n*	WHO FC	mRAP (*mm Hg*)	CI (L/min/m^2^)	SvO_2_ (*%*)	RVEF (*%*)	RVESVi (*% Predicted*)
1	267	3.6 ± 0.5	12 (8, 16)	2.04 (1.67, 2.65)	58 (52, 66)	33 (27–42)	283 (225, 405)
2	206	3.3 ± 0.5	10 (6, 15)	2.31 (1.80, 2.86)	61 (54, 67)	32 (25–44)	277 (163, 338)
3	196	3.1 ± 0.5	10 (6, 14)	2.28 (1.81, 2.90)	62 (55, 69)	35 (24, 48)	253 (159, 371)
4	172	3.0 ± 0.4	9 (6, 13)	2.40 (1.89, 2.9)	64 (58, 68)	35 (28, 43)	240 (162, 328)
5	137	2.9 ± 0.5	8 (6, 13)	2.50 (2.00, 3.05)	66 (59, 70)	34 (25, 44)	237 (152, 328)
6	110	2.7 ± 0.5	8 (5, 11)	2.60 (2.17, 3.20)	68 (63, 71)	36 (25, 48)	176 (132, 264)
7	69	2.6 ± 0.5	9 (6, 12)	2.56 (2.20, 3.17)	66 (59, 71)	42 (34, 50)	183 (122, 258)
8–12	83	2.4 ± 0.5	7 (5, 9)	2.85 (2.22, 3.22)	69 (63, 72)	40 (27, 48)	159 (120, 241)

*Definition of abbreviations*: CI = cardiac index; ISWT = incremental shuttle walking test; mRAP = mean right atrial pressure; MRI = magnetic resonance imaging; RVEF = right ventricular ejection fraction; RVESVi = right ventricular end-systolic volume (indexed for body surface area and corrected for age and sex); SvO_2_ = mixed venous oxygen saturation; WHO FC = World Health Organization functional class.

Data are displayed as mean ± standard deviation or median (first quartile, third quartile).

**Figure 2.**
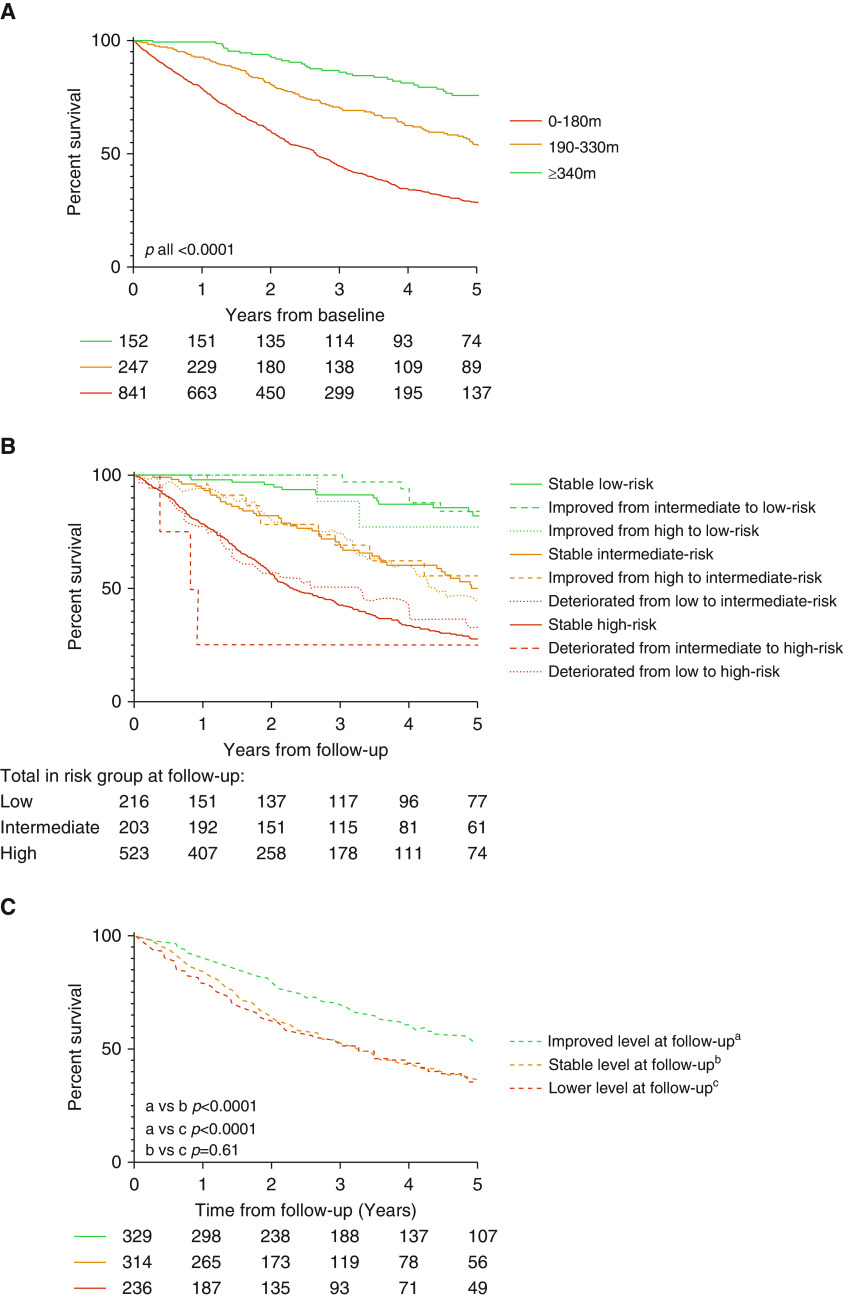
Kaplan-Meier survival curves for (*A*) incremental shuttle walking test distance risk groups at baseline, (*B*) transition of incremental shuttle walking test distance risk groups between baseline and follow-up, and (*C*) comparison of patients who, at follow-up, achieved at least one higher incremental shuttle walking test level, achieved the same incremental shuttle walking test level, or achieved a lower level than at baseline.

At the follow-up ISWT, the thresholds accurately identified patients at low, intermediate, and high risk in the combined IPAH and PAH-CTD cohort (1-yr survival of 97%, 94%, and 78%, respectively) and in the individual disease groups. Kaplan-Meier graphs showing 5-year transplant-free survival at baseline and follow-up and demonstrating risk transition are displayed in Figure 2.

### Age <50 Years

Using the above thresholds in incident patients aged <50 years, 30% were identified as low risk and had 0% 1-year mortality. Seventy patients (28%) were intermediate risk, in whom the observed 1-year mortality was lower than expected at 3%, whereas 42% of patients were high risk and had a 1-year mortality of 15%. A scatterplot showing baseline and follow-up distances and 1-year mortality is shown in Figure E1.

### Treatment Response

**Figure 3.**
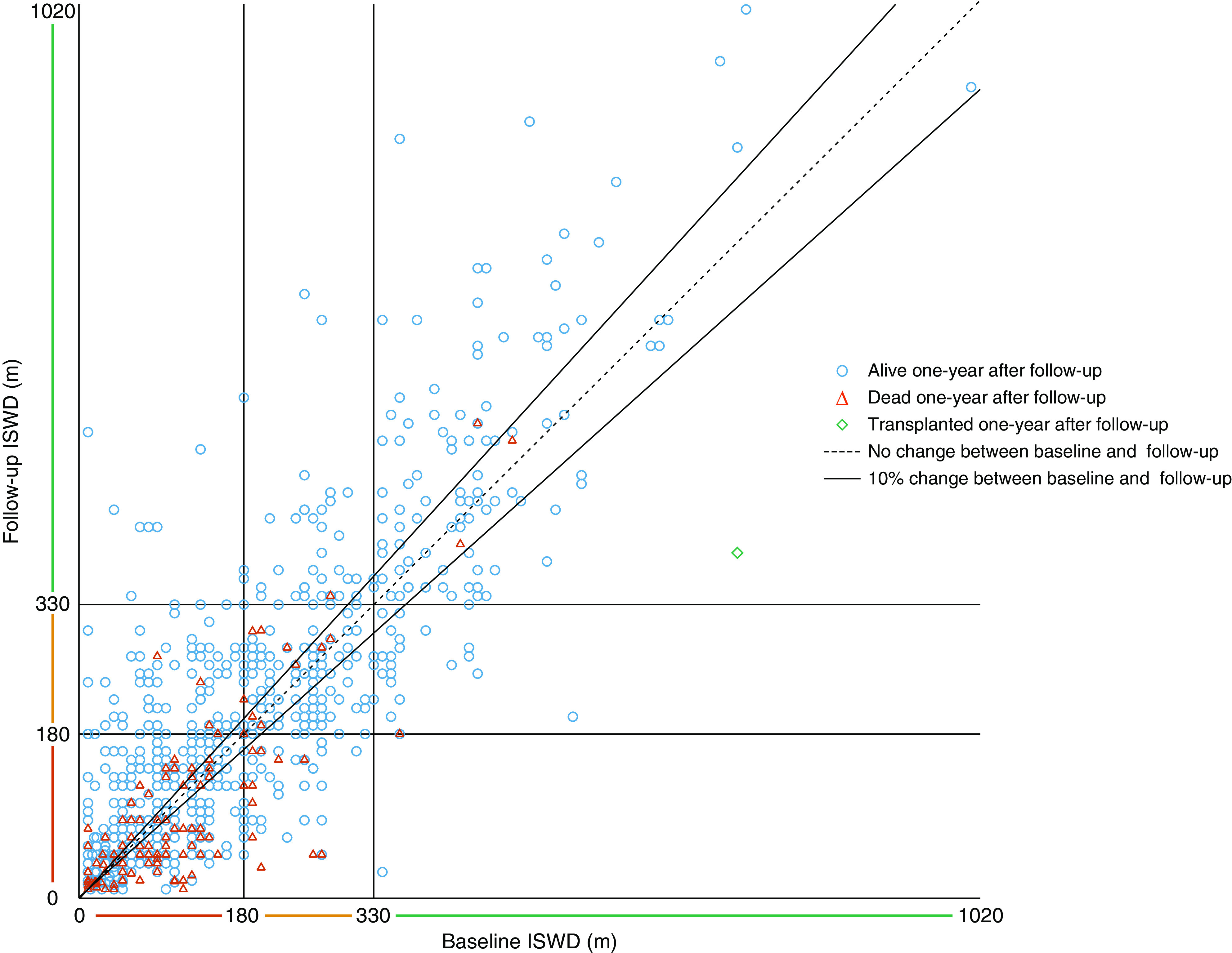
A scatterplot showing individual baseline and follow-up incremental shuttle walking test distances and mortality or transplant within 1-year of follow-up incremental shuttle walking test distance in patients with idiopathic pulmonary arterial hypertension and pulmonary arterial hypertension due to connective tissue disease (*n* = 879). ISWD = incremental shuttle walking test distance.

The baseline median ISWD was 110 m (40, 220), and paired tests at follow-up were available for 879 patients. At follow-up, 132 (15%) patients had improved their ISWT risk category (i.e., had improved to either intermediate or low-risk distance), and 83 (9%) patients had deteriorated. A scatterplot demonstrating individual baseline and follow-up distance is displayed in Figure 3. At paired testing, a median improvement of +10 m (−30, 50; *P* < 0.0001) was seen overall. Patients who achieved at least one ISWT level higher than at baseline (*n* = 329 [37%]), and therefore achieved a higher velocity, had significantly better 1- and 5-year survival (90% and 54%, respectively) than those who either remained in the same level (*n* = 314 [36%]; 1- and 5-yr survival 84% and 37%, respectively; *P* < 0.0001) or deteriorated (*n* = 236 [27%]; 1- and 5-yr survival 79% and 36%, respectively; *P* < 0.0001), whereas there was no significant survival difference between those who were stable or deteriorated (*P* = 0.61; Figure 2).

### Use in Conjunction with Risk Stratification Scores

**Figure 4.**
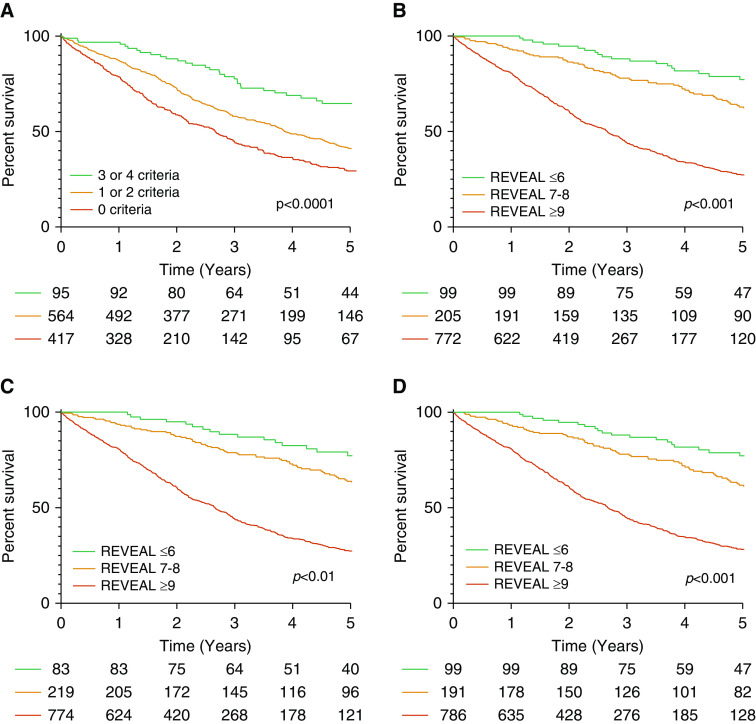
Kaplan-Meier analysis demonstrating survival in (*A*) French Pulmonary Hypertension Registry low-risk approach; (*B*) REVEAL (Registry to Evaluate Early and Long-Term Pulmonary Arterial Hypertension Disease Management) 2.0 variation 1 (incremental shuttle walking test distance [ISWD] 0–180 m = +1 point, 190–330 m = 0 points, 340–420 m = −1 point, and ≥430 m = −2 points); (*C*) REVEAL 2.0 variation 2 (ISWD 0–180 m = +1 point, 190–330 m = 0 points, and ≥340 m = −1 point); and (*D*) REVEAL 2.0 variation 3 (ISWD 0–30 m = +2 points, 40–180 m = +1 point, 190–330 m = 0 points, 340–420 m = −1 point, and ≥430 m = −2 points).

Patients with baseline RHC data available including mean right atrial pressure and cardiac index (*n* = 1,076) were selected to assess whether ISWD thresholds could be used in conjunction with other risk stratification scores in place of 6MWD thresholds. For the French Pulmonary Hypertension Registry (FPHR) low-risk invasive approach to the ESC/ERS guidelines, a low-risk 6MWD of >440 m was substituted with a low-risk ISWD of ≥340 m. Survival differed significantly based on the number of low-risk criteria (0–4) between all groups (*P* < 0.05) and at receiver operating characteristic analysis produced a *C*-statistic of 0.61 (95% confidence interval [CI], 0.57–0.66), which was unchanged when used in the IPAH group in isolation and higher than when the FPHR approach was used without any walking test (*C*-statistic, 0.59; 95% CI, 0.55–0.64). Kaplan-Meier analysis for an abbreviated three-category risk score (three or four criteria = low risk, one or two criteria = intermediate risk, and 0 criteria = high risk) is displayed in Figure 4A and demonstrates the separation of curves for each risk category (*P* < 0.0001 for all). Using this three-category FPHR risk score, low- and high-risk groups were accurately identified (1-yr survival 96% and 78%, respectively) but risk in the intermediate group was underestimated (1-yr survival 87%).

**Figure 5.**
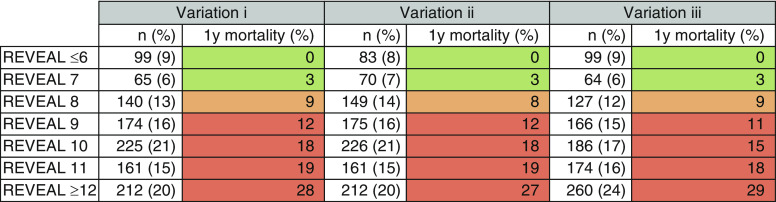
Risk of mortality by REVEAL (Registry to Evaluate Early and Long-Term Pulmonary Arterial Hypertension Disease Management) 2.0 score, using variations of REVEAL 2.0 incorporating incremental shuttle walking test distance (ISWD) as follows: REVEAL 2.0 variation 1 (ISWD 0–180 m = +1 point, 190–330 m = 0 points, 340–420 m = −1 point, and ≥430 m = −2 points), REVEAL 2.0 variation 2 (ISWD 0–180 m = +1 point, 190–330 m = 0 points, and ≥340 m  = −1 point), and REVEAL 2.0 variation 3 (ISWD 0–30 m = +2 points, 40–180 m = +1 point, 190 = 330 m = 0 points, 340–420 m = −1 point, and ≥430 m = −2 points).

When assessing the REVEAL 2.0 score in the same population, three variations for substituting 6MWD with ISWD were derived based on *1*) thresholds similar to the 6MWD thresholds used in REVEAL 2.0; *2*) thresholds of low, intermediate, and high risk identified at baseline; and *3*) thresholds of low, intermediate, and high risk identified at baseline with an extra point addition or deduction for very high (≤30 m) and very low risk (≥430 m), respectively, derived from the baseline data shown in Table 1. The REVEAL 2.0 *C*-statistic for 1-year mortality without a walking test was 0.66 (95% CI, 0.62–0.70); including ISWD thresholds from variation *3* produced a *C*-statistic of 0.71 (95% CI, 0.67–0.75), compared with 0.69 for variations *1* and *2*. Low- (≤6), intermediate- (7 and 8), and high-risk (≥9) REVEAL 2.0 scores (scores grouped as previously described) (28) accurately predicted 1-year mortality; survival curves are displayed in Figure 4, and detailed analysis of 1-year mortality for REVEAL 2.0 scores are displayed in Figure 5. In all variations, patients with a REVEAL 2.0 score ≤6 had a 0% 1-year mortality, and patients with a REVEAL 2.0 score of ≥9 had a 1-year mortality of 19–20%.

## Discussion

In a large cohort of patients with IPAH and PAH-CTD, we have demonstrated that routine use of maximal exercise testing can risk-stratify patients into low, intermediate, and high risk of 1-year mortality/lung transplantation. Using a three-level risk score, we have identified ISWT thresholds at baseline, shown the clinical use in conjunction with other risk stratification scores, and demonstrated that thresholds identified at baseline risk-stratify patients at follow-up.

Exercise capacity is recognized as an important physiological marker in PAH, and as a validated measure, the 6MWT has been the mainstay of exercise testing in PAH both in routine practice and in clinical trials (9). In early studies assessing PAH therapies, 6MWD was demonstrated to be a marker of treatment response (29). Absolute distances are prognostic, and the deterioration of 6MWD is strongly associated with poor prognosis (15, 30). Despite this, there has been criticism of the 6MWT, particularly regarding its role as an endpoint in clinical trials (13, 31), as prospective and retrospective studies have been unable to demonstrate that improvements in 6MWD are independently associated with survival (15, 30). Furthermore, it is a submaximal test and may suffer from a ceiling effect, potentially limiting its use in younger patients or those with mild disease (32).

We have previously shown that as an alternative but maximal field walking test, the ISWT provides a measure of maximal exercise capacity without a ceiling effect and can identify exercise limitation in asymptomatic patients diagnosed with pulmonary hypertension in World Health Organization functional class I (20, 33). Using data from the present study, we have now identified that maximal exercise testing using the ISWT can risk-stratify patients with IPAH and PAH-CTD. At baseline, in incident and treatment-naive patients, levels of the ISWT demonstrated good separation for both 1-year and longer-term survival. As a risk stratification tool, thresholds established at baseline were applicable at follow-up. As has been demonstrated with other prognostic investigations and risk stratification tools, patients who improved their risk profile demonstrated comparable longer-term survival with patients originally displaying that level of risk (24, 34, 35).

A drawback of the 6MWT is that it suffers from a ceiling effect, whereby patients who walk >450 m at baseline may not improve their walking distance in response to treatment despite improvements in World Health Organization functional class and hemodynamics (20). In this study we have shown that even among patients who walked ≥340 m at baseline and remained in the low-risk group at follow-up, 63% improved absolute ISWD in response to treatment. At higher follow-up distances of ≥430 m and ≥530 m, 68% and 69% of patients, respectively, were able to improve their ISWD after commencing treatment. A further criticism of the 6MWT is that younger patients with severe PAH, and therefore at high risk of mortality, may still be able to walk distances >500 m (12). We have therefore undertaken an exploratory analysis on patients aged <50 years and identified no mortality at 1 year for patients with a low-risk ISWD of ≥340 m.

We have also demonstrated that patients who were able to achieve a higher ISWT level had significantly better long-term survival than patients who either remained in the same level or achieved a lower level at follow-up. This is expected, as each level of the ISWT requires a higher maximal walking or running velocity, which has been shown to correlate with maximal oxygen intake (peak VO_2_) in other cardiorespiratory diseases (18, 19, 36). Peak VO_2_ has been identified as a strong prognostic marker of survival in PAH when measured by incremental CPET (37), and other centers have confirmed the value of incremental exercise testing in the assessment of patients with pulmonary hypertension (38).

Associations between incremental exercise testing and hemodynamics have been shown previously, and we have expanded on this by showing the association between this incremental test and important prognostic parameters from cardiac magnetic resonance imaging with a stepwise reduction in right ventricular end-systolic volume percentage predicted with each level of the ISWT (20, 35, 38).

Our data demonstrate that ISWT thresholds can now be considered for incorporation into widely used risk stratification tools. Using the French low-risk invasive approach to risk stratification, substitution of the 6MWT distances with equivalent distances for low, intermediate, and high risk from the ISWT continued to show five distinct risk groups at survival analysis. When combined into a three-category risk score, patients at low (three or four criteria) and high risk (zero criteria) had a 1-year mortality of 4% and 22%, respectively. Boucly and colleagues noted the difficulties of defining an intermediate-risk group, and we found that the presence of one or two low-risk criteria underestimated 1-year mortality, which was also seen when this approach was applied to the REVEAL population (25, 28). The *C*-statistic of 0.61 in our population of patients with IPAH is similar to that identified when the French approach was tested in the REVEAL registry (0.62), although no *C*-statistic is provided in the original research (25, 28).

In the REVEAL 2.0 risk score, we have shown that when 6MWT distances are substituted with variations of ISWT thresholds, a three-level risk score accurately predicts 1-year mortality in this population. The *C*-statistic of 0.71 is lower than that identified in REVEAL 2.0 (0.76), and this may be the result of a phenotypically different PAH population. In our study, we included only patients with IPAH and PAH-CTD rather than other forms of PAH such as congenital heart disease. Furthermore, in our study, risk stratification approaches were applied to treatment-naive patients rather than a mixture of incident and prevalent patients as in the REVEAL study. These factors, particularly the absence of patients with congenital heart disease–related PAH (the presence of which scores −2 points in REVEAL 2.0), may also explain why a relatively small number of our patients were identified as being at low risk by REVEAL 2.0 when compared with the original study and external validation studies (28, 39).

### Limitations

Distances achieved on the 6MWT and ISWT are not directly comparable, and the thresholds used in this study were identified from baseline data. Although we have assessed and confirmed that these thresholds remain valid at follow-up, as in any single-center study, both the thresholds and their role in risk stratification tools require prospective validation in a separate population. Although we are unable to directly compare sensitivity and specificity for the 6MWT and ISWT thresholds in the same population, our data support the use of the ISWT as a tool in risk stratification in PAH. In this study, we have focused on patients with IPAH and PAH-CTD, and further work is required to assess whether these thresholds remain valid in patients with other forms of PAH. All-cause mortality or transplantation was used as the primary endpoint, and patients may have died of causes unrelated to PAH. Follow-up data were unavailable for 29% of patients, although a proportion of these patients did not survive to follow-up (15.4% died within 1 yr of diagnosis). These missing data do not include patients who attended the clinic but were unable to perform the ISWT because of breathlessness, as these patients were assigned a distance of 0 m. Finally, although the thresholds identified a large proportion of patients at high risk of 1-year mortality, this may reflect a high-risk population as demonstrated by the large number of patients with a high REVEAL 2.0 score, with a corresponding 1-year mortality of approximately 20%. The ISWT is simple to perform and, in contrast to the 6MWT, requires a 10-m rather than 30-m corridor, with an average time to complete the test of approximately 3 minutes, making it straightforward to incorporate into clinical practice.

### Conclusions

Maximal exercise testing can be used to risk-stratify patients with pulmonary hypertension, including IPAH and PAH-CTD, and this study supports the routine use of maximal exercise testing in conjunction with other risk stratification tools. This study highlights the potential value of the ISWT as an alternative to the 6MWT, combining some of the advantages of maximal exercise testing and maintaining the simplicity of a simple-to-perform field test.
